# Transcriptional, Post-Transcriptional, and Post-Translational Mechanisms Rewrite the Tubulin Code During Cardiac Hypertrophy and Failure

**DOI:** 10.3389/fcell.2022.837486

**Published:** 2022-04-01

**Authors:** Sai Aung Phyo, Keita Uchida, Christina Yingxian Chen, Matthew A. Caporizzo, Kenneth Bedi, Joanna Griffin, Kenneth Margulies, Benjamin L. Prosser

**Affiliations:** ^1^ Department of Genetics and Epigenetics, University of Pennsylvania Perelman School of Medicine, Philadelphia, PA, United States; ^2^ Department of Physiology, Pennsylvania Muscle Institute, University of Pennsylvania Perelman School of Medicine, Philadelphia, PA, United States; ^3^ Department of Medicine, Cardiovascular Institute, University of Pennsylvania Perelman School of Medicine, Philadelphia, PA, United States

**Keywords:** hypertrophy, heart failure, tubulin isoforms, transcription, autoregulation

## Abstract

A proliferated and post-translationally modified microtubule network underlies cellular growth in cardiac hypertrophy and contributes to contractile dysfunction in heart failure. Yet how the heart achieves this modified network is poorly understood. Determining how the “tubulin code”—the permutations of tubulin isoforms and post-translational modifications—is rewritten upon cardiac stress may provide new targets to modulate cardiac remodeling. Further, while tubulin can autoregulate its own expression, it is unknown if autoregulation is operant in the heart or tuned in response to stress. Here we use heart failure patient samples and murine models of cardiac remodeling to interrogate transcriptional, autoregulatory, and post-translational mechanisms that contribute to microtubule network remodeling at different stages of heart disease. We find that autoregulation is operant across tubulin isoforms in the heart and leads to an apparent disconnect in tubulin mRNA and protein levels in heart failure. We also find that within 4 h of a hypertrophic stimulus and prior to cardiac growth, microtubule detyrosination is rapidly induced to help stabilize the network. This occurs concomitant with rapid transcriptional and autoregulatory activation of specific tubulin isoforms and microtubule motors. Upon continued hypertrophic stimulation, there is an increase in post-translationally modified microtubule tracks and anterograde motors to support cardiac growth, while total tubulin content increases through progressive transcriptional and autoregulatory induction of tubulin isoforms. Our work provides a new model for how the tubulin code is rapidly rewritten to establish a proliferated, stable microtubule network that drives cardiac remodeling, and provides the first evidence of tunable tubulin autoregulation during pathological progression.

## Introduction

Heart Failure (HF) is a complex pathological condition in which cardiac performance fails to match systemic demand. HF is commonly preceded by an enlargement of the heart known as cardiac hypertrophy, which serves as a major risk factor for progression to HF. As such, understanding the molecular determinants of hypertrophy may reveal novel targets for HF prevention.

Microtubules are hollow tubes formed from the polymerization of α- and β- tubulin dimers that play essential roles in the structural support of cells, intracellular transport, and cell division. They exhibit stochastic growth and shrinkage and maintain a dynamic equilibrium between free and polymerized tubulin (Supplementary Figure S1A). Through their trafficking role, microtubules regulate cardiomyocyte electrical activity, mitochondrial dynamics, protein degradation and local translation, while also forming load-bearing structures that influence myocyte mechanics and mechano-signaling (Caporizzo et al., 2019).

During cardiac hypertrophy and heart failure, the microtubule network is significantly remodeled and acts as a double-edged sword. On one hand, a proliferated, stable microtubule network is essential for the development of cardiac hypertrophy in response to stressors such as adrenergic stimulation and hemodynamic overload (Sato et al., 1997; Fassett et al., 2009; Fassett et al., 2019; Scarborough et al., 2021). Upon such hypertrophic stimuli, a dense microtubule network and the anterograde motor protein kinesin-1 coordinates the trafficking of mRNA and the translational machinery to control local synthesis and integration of nascent proteins (Scarborough et al., 2021). In the absence of microtubules, increased protein translation is decoupled from protein integration and the heart fails to grow (Scarborough et al., 2021), identifying an essential role of microtubule-based transport in adaptive cardiac growth.

Yet upon chronic stress, the densified microtubule network can also contribute to contractile dysfunction in HF(Tsutsui et al., 1999; Caporizzo et al., 2018; Chen et al., 2018). A collective body of research has established a causal link between aberrant microtubule network remodeling and impaired cardiac mechanics in HF. Tubulin mass, and consequently microtubule network density, is consistently increased in the myocardium of HF patients (Chen et al., 2018; Schuldt et al., 2021) and pressure-overloaded animals (Sato et al., 1997; Fassett et al., 2019), and its destabilization can improve dysfunctional cardiac mechanics (Tsutsui et al., 1993; Cheng et al., 2008; Chen et al., 2018; Caporizzo et al., 2020).

**FIGURE 1:**
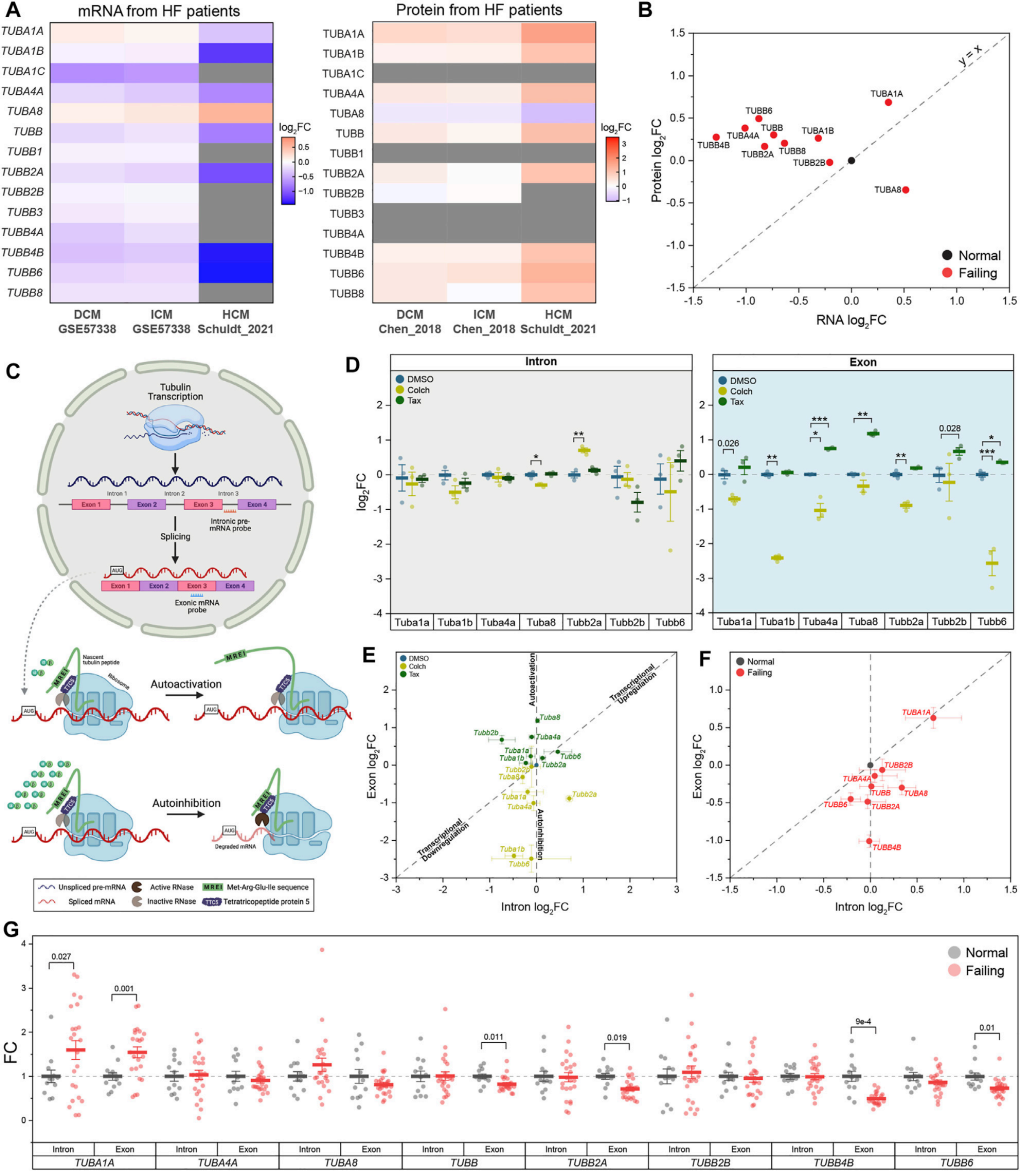
Tubulin autoregulation is operant in the heart and activated in heart failure. **(A)** Heatmaps of previously published mRNA (left) & protein (right) data of human αβ-tubulin isoforms during dilated (DCM) (Liu et al., 2015, Chen et al., 2018), ischemic (ICM) (Liu et al., 2015, Chen et al., 2018), & hypertrophic (HCM) (Schuldtet al., 2021) cardiomyopathies. **(B)** Scatter-plot of log_2_fold-change of mRNA on x-axis and log_2_fold-change of protein on y-axis in heart failure; each data point represents an average log_2_fold-change value from DCM and ICM groups from **(A)**, and y = x represents a proportionate change between mRNA and peptide. **(C)** Schematic of tubulin autoregulation: The introns of tubulin pre-mRNA are spliced out and the mRNA is fully translated in the absence of excess free tubulin; in the presence of excess free tubulin, the mRNA, but not the pre-mRNA, is degraded. **(D)** Relative log_2_fold-change of mRNA counts of intronic (left) and exonic (right) αβ-tubulin isoforms in isolated adult mouse cardiomyocytes after treatment with either depolymerizing (Colch) or polymerizing (Tax) agents (*n* = 3); whiskers represent ± 1SEM, bolded line represents mean, * represents p-value from Welch-corrected two-tailed two-sample t-test on non-log data <0.025 (Bonferroni-corrected for two comparisons), ** represents*p* < 0.01, and *** represents *p* < 0.001. **(E)** Scatter-plot of relative log_2_fold-change of intron on x-axis and exon on y-axis after Colch or Tax treatment in adult mouse cardiomyocytes; whiskers represent ± 1SEM. **(F)** Scatter-plot of relative log_2_fold-change of intron on x-axis and exon on y-axis in near-normal and failing patient heart samples; whiskers represent ± 1SEM. **(G)** Relative fold-change of mRNA counts of intronic and exonic αβ-tubulin isoforms in near-normal and failing patient heart samples; whiskers represent ± 1SEM, bolded line represents mean, and p-values are from Welch-corrected two-tailed two-sample t-test.

While the state of the microtubule network in advanced HF has been well-defined by recent studies (Chen et al., 2018; Schuldt et al., 2020), we know little about the drivers and temporal progression of changes to the microtubule network that occur during cardiac remodeling. A seemingly obvious mechanism to increase tubulin mass is transcriptional upregulation; yet when we examine published transcriptomic and proteomic data from HF samples, we observe a surprising but consistent inverse correlation between tubulin mRNA and protein levels across different causes of HF in multiple studies (Figures 1A,B). This motivates a deeper examination between transcriptional and translation coupling of tubulin isoforms and other factors that could contribute to microtubule proliferation.

There are a multitude of α and β tubulin isoforms that arise from alternative tubulin genes; in humans, there are nine α and nine β -tubulin isoforms, and in mice, seven α and eight β isoforms (Supplementary Figure S1B). The abundance of tubulin transcripts can be controlled through autoregulation, a tubulin-specific mRNA rheostat in which an excess of free tubulin can activate a ribosomal RNase to degrade nascent tubulin transcripts (autoinhibition); conversely, if free tubulin levels are reduced, autoregulation is released (autoactivation) to promote tubulin synthesis and restore free tubulin content (Gasic and Mitchison 2019) (Figure 1C). The extent to which tubulin isoforms are controlled through transcriptional or autoregulatory mechanisms has not been characterized, and autoregulation has not been examined in any capacity in the heart. Finally, any pathological relevance of autoregulation in cardiac or other tissues is largely unexplored.

The stabilization (i.e., protection from breakdown) of polymerized microtubules is another potentially important driver of the dense microtubule network observed in hypertrophy and HF. Microtubules are stabilized through association with microtubule-associated proteins (MAPs) and motors as well as through post-translational modifications (PTMs) of tubulin (Supplementary Figure S1). Acetylation of polymerized α-tubulin produces long-lived and resilient microtubules that are resistant against repeated mechanical stresses (Kalebic et al., 2013; Portran et al., 2017), while detyrosination - the removal of a tyrosine residue on the C-terminal tail of α-tubulin by vasohibins 1 and 2 (VASH1/2) (Aillaud et al., 2017; Nieuwenhuis et al., 2017)—stabilizes microtubules by modulating their interactions with depolymerizing effector proteins (Peris et al., 2009; Chen et al., 2021). The permutations of PTMs and tubulin isoforms is known as the “tubulin code” (Supplementary Figure S1), which creates microtubule networks with distinct biochemical and mechanical properties. Altered detyrosination (Chen et al., 2018; Yu et al., 2021), acetylation (Swiatlowska et al., 2020), and MAP (Cheng et al., 2010; Li et al., 2018; Yu et al., 2021) binding are each implicated in pathological cardiac remodeling; yet how the tubulin code is rewritten during cardiac hypertrophy and HF remains largely unclear.

In this study, we interrogate changes to the tubulin code, MAPs, and motors at discrete stages of pathological cardiac remodeling. We find that surprisingly rapid and isoform-specific transcriptional induction and autoactivation of tubulin mRNA combine with post-translational detyrosination to drive microtubule stabilization and proliferation during early cardiac growth. We also find that in progressed heart failure, there is a switch to autoinhibition that reduces tubulin mRNA expression in the face of elevated tubulin protein content. This work identifies roles for autoregulation in rewriting the tubulin code during cardiac remodeling and may inform on approaches intended to modulate the course of hypertrophy and its progression to HF.

## Methods

### Human Myocardial Tissue

Procurement of human myocardial tissue was performed under protocols and ethical regulations approved by Institutional Review Boards at the University of Pennsylvania and the Gift-of-Life Donor Program (Pennsylvania, United States) and as described (Chen et al., 2018). In summary, failing human hearts were procured at the time of orthotropic heart transplantation at the Hospital of the University of Pennsylvania following informed consent from all participants. Non-failing hearts were obtained at the time of organ donation from cadaveric donors. In all cases, hearts were arrested *in situ* using ice-cold cardioplegia solution and transported on wet ice. Transmural myocardial samples were dissected from the mid left ventricular free wall below the papillary muscle and the samples were kept frozen at 80°C. Contractile parameters, including left ventricle ejection fraction, were determined by echocardiography in subjects. In this study, a total of 35 donor hearts were used. 12 donors were classified as near-normal non-failing (NF) without left-ventricular hypertrophy, and 23 donors were classified as heart failure with 12 hearts from hypertrophic cardiomyopathy patients and 11 hearts from dilated cardiomyopathy patients.

### Animal Care

Animal care and procedures were approved and performed in accordance with the standards set forth by the University of Pennsylvania Institutional Animal Care and Use Committee (IACUC) and the Guide for the Care and Use of Laboratory Animals published by the US National Institutes of Health (NIH).

### Drug Injection

Eight to 12 weeks old male C57/Bl6 mice were used throughout the study. On days 0 and 2, based on their body weights, mice were subcutaneously injected with either ascorbic acid (Ctrl, Sigma-Aldrich: A92902), 10 mg/kg phenylephrine (PE, Sigma-Aldrich: P6126) prepared in Ctrl, or 5 mg/kg isoproterenol (Iso, Sigma-Aldrich: I6504) prepared in Ctrl.

### Cardiac Tissue Harvest

Mice were put under general anesthesia using isoflurane and the hearts were surgically removed. Excised hearts were thoroughly washed in ice-cooled PBS and extra-cardiac tissues were removed. To properly measure heart weight (HW), residual blood from the chambers was removed by sandwiching the heart between Kimwipes and gently squeezing it. After HW measurement, atrial and right ventricular tissues were removed, the remaining septal and left-ventricular tissues were cut into five pieces of similar size and from similar locations of the heart. The weights of the individual pieces were recorded, frozen in liquid nitrogen, and stored at -80°C until further processing. Concurrent with tissue harvest, the tibia length (TL) of respective mouse was measured to calculate HW-over-TL (HW/TL).

### Exclusion Criteria

During the study: for the 4-h time point, there were six mice per treatment group for a total of 18 mice, and for the 4-days time point, there were eight mice per treatment group for a total of 24 mice. As we aimed to study mice who underwent consistent cardiac hypertrophy, for the 4-days time point, we set exclusion criteria as the followings: (1) hearts whose HW/TL were beyond 2 SD of the population mean, and (2) experimental hearts whose classical hypertrophy response genes were not changed relative to that of the control hearts. After the removal of outliers, in the final study: for the 4-h time point, there are six mice per treatment group for a total of 18 mice, and for the 4-days time point, there are seven mice in Ctrl, seven mice in PE, and six mice in Iso, for a total of 20 mice.

### Mouse Cardiomyocyte Isolation, Culture, and Drug Treatment

Primary adult ventricular myocytes were isolated from eight- to 12-week-old C57/Bl6 mice using the protocol previously described (Prosser et al., 2011). Briefly, mice were put under general anesthesia using isoflurane and were injected peritoneally with heparin (∼1,000 units/kg). The heart was excised and cannulated to a Langendorff apparatus for retrograde perfusion with enzymatic digestion solution at 37°C. Once digested, the heart was minced and triturated with glass pipettes. The isolated cardiomyocytes were centrifuged at 300 revolution per minute for 2 min. The supernatant containing debris was discarded and the isolated cells were resuspended in cardiomyocyte media containing Medium 199 (GIBCO: 11150-59) supplemented with 1x insulin-transferrin-selenium-X (GIBCO: 51500-56), 20 mM HEPES pH 7.4, 0.1 mg/ml Primocin, and 25 μmol/L of cytochalasin D. Immediately following cell isolation, the cardiomyocytes were treated with either DMSO, 10 μM colchicine, or 10 μM taxol, and incubated at 37°C and 5% CO_2_ for 6 h.

### Echocardiography

On day 4, transthoracic echocardiography was performed on mice, which were anesthetized using intraperitoneal injection of 0.01 ml/G body-weight of 2.5% Avertin, using Vevo2100 Ultrasound System (VisualSonics Inc., Toronto, Ontario, Canada). Fractional shortening, chamber dimensions, and ventricular wall-thickness were measured from short axis M-mode images at the mid-level view of the papillary muscle.

### Total Protein Lysate Preparation

Frozen aliquoted cardiac tissue obtained from similar locations of the heart was pulverized finely using a liquid nitrogen-cooled mortar and pestle. 1x Radioimmunoprecipitation assay (RIPA) buffer (Cayman Chemical Company: 10010263) supplemented with 1x protease inhibitor cocktail (Cell Signaling Technology: 5872S) and 1:200 diluted endonuclease (Lucigen: OC7850K) was immediately added to the pulverized tissue at a constant ratio of 15 μL/mg of tissue. The sample was then mechanically homogenized using a handheld homogenizer until visible chunks of tissues were dissociated. The sample was incubated for 10 min on ice to allow endonuclease to cleave DNA. After processing of all samples, the samples underwent two freeze-thaw cycles, after which, equal-volume of 5% SDS-10% glycerol boiling (SGB) buffer was added to each sample. The samples were vortexed thoroughly then heated to 100°C for 8 min. Residual undissolved cell debris were removed from the resulting samples by centrifugation at 8000 g for 5 min at room temperature (22°C). The concentrations of the total protein were determined using Bicinchoninic acid (BCA) assay; all samples were diluted to 4 μg/μL using RIPA:SGB buffer. The diluted total protein lysates were aliquoted and stored at −80°C until further processing.

### Microtubule Fractionation

100 mM PIPES-KOH pH 6.8, 1 mM MgCl_2_, 1 mM EGTA-KOH pH 7.7 (PME) buffer was prepared fresh and was supplemented with 1 mM DTT, 1 mM GTP (Sigma-Aldrich: G8877), and 1x protease inhibitor cocktail. Seven parts supplemented PME buffer was mixed with three parts glycerol; the final PME-30G buffer was kept incubated in a 37°C water bath. Frozen aliquoted cardiac tissue obtained from similar locations of the heart was pulverized crudely using a liquid nitrogen-cooled mortar and pestle. Immediately following pulverization, warmed PME-30G buffer was added at a constant ratio of 20 μL/mg of tissue. The sample was then mechanically homogenized using the handheld homogenizer until visible chunks of tissues were dissociated and was set aside at 22°C until all samples were processed. All processed samples were then centrifuged at 16000 g for 15 min at 30°C; the supernatants were transferred into fresh tubes and were saved as free tubulin (Free) fractions. 10 μL of 1 part RIPA and 1 part SGB (RIPA:SGB) buffer was added to the pellet obtained from 1 mg of tissue and the sample was homogenized using the handheld homogenizer. After processing of all samples, the samples were heated to 100°C for 8 min, cooled on ice, and centrifuged at 8000 g for 5 min at 22°C; the supernatants were transferred into fresh tubes and were saved as polymerized tubulin (Poly) fractions. The concentrations of the Poly fractions were determined using BCA assay. The Poly fractions were diluted to 4 μg/μL using RIPA:SGB buffer; the respective Free fraction was diluted with PME-30G buffer using twice the volume needed to dilute the Poly fraction. The final diluted Free and Poly fractions were aliquoted and stored at −80°C until further processing.

### Sample Preparations and Western Blot Analysis

To quantify the relative abundance of specific proteins of interest in the total protein lysate, aliquoted diluted total protein lysate samples were thawed at 22°C. One part 4x loading buffer (125 mM Tris-HCl pH 6.8, 35% v/v glycerol, 0.2% w/v Orange G) freshly supplemented with 10% v/v β-mercepthoethanol (BME) was mixed with three parts total protein lysate to get final concentrations of 1x loading buffer with 2.5% BME, and 3 μg/μL of total protein. The final samples were heated to 100°C for 8 min. The heated samples were cooled to 22°C, centrifuged briefly, vortexed thoroughly, and loaded 5μL/sample onto precast protein gels (Bio-Rad: 5671085).

To quantify the relative abundances of the Free and Poly fractions, aliquoted diluted Free and Poly fractions were thawed at 22°C. For the Free fractions, 2x loading buffer (62.5 mM Tris-HCL pH 6.8, 5% v/v SDS, 0% glycerol, 0.1% w/v Orange G) freshly supplemented with 5% v/v BME was used, whereas, for the Poly fractions, 4x loading buffer freshly supplemented with 10% v/v BME was used; to prepare the final samples, the respective loading buffers were diluted to 1x using the Free and Poly fractions. The final samples were heated to 100°C for 8 min. The heated samples were cooled to 22°C, centrifuged briefly, vortexed thoroughly, and loaded 5 μL/Poly fraction and 10 μL/Free fraction onto precast protein gels.

Protein gel electrophoresis was carried out under constant voltage of 135 V for the Midi gels for 1 h. The resolved proteins were transferred onto a nitrocellulose membrane using the Turbo Transfer System (Bio-Rad) under recommended conditions. The post-transferred membrane was blocked in blocking buffer (LI-COR Biosciences: 927-60003) for at least 1 h at 22°C (or overnight at 4°C). The blocked membrane was incubated overnight at 4°C with primary antibodies diluted in 1x Tris buffered saline with Tween-20 (TBST, Cell Signaling Technology: 9997S). The membrane was washed twice using TBST, and incubated for 1 h at 22°C with secondary antibodies diluted in blocking buffer. The final immunoblotted membrane was washed twice using TBST and was imaged using the Odyssey Western Blot Imaging System (LI-COR Biosciences).

### WB Data Analysis

The WB data was analyzed using Image Studio Lite (LI-COR Biosciences). The signal intensity of an individual band was obtained by drawing a rectangular block encompassing the entire band. The background was thresholded using the parameters: median, border width = 3, Top/Bottom. Two technical replicates (n) per sample, and six biological replicates (N) per treatment for 4-h time point and eight biological replicates per treatment for 5-days time point were used in the analysis. GAPDH intensity was used as a loading control. A mean value of the Ctrls that were run on the same blot was used to normalize the data and to calculate the relative fold-changes over the Ctrl. Statistical analyses were performed as described below.

### Primary and Secondary Antibodies

(see table below)

**Table:** 

Target	Vendor	Host species	Clonal	Product no	Concentration used in WB
Total α-tubulin	Abcam	Mouse	Mono	ab7291	1:3000
Total α-tubulin	Abcam	Rabbit	Poly	ab4074	1:2000
Total β-tubulin	Abcam	Rabbit	Poly	ab6046	1:1500
Acetylated α-tubulin	Abcam	Mouse	Mono	ab24610	1:1000
Detyrosinated α-tubulin	Abcam	Rabbit	Poly	ab48389	1:1000
Polyglutamylated α-tubulin	Adipogen	Mouse	Mono	50-436-394	1:500
Δ2 α-tubulin	Moutin Lab	Rabbit	Poly		1:5000
Polyglycylated tubulin	EMD Millipore	Mouse	Mono	MABS276	1:700
Kif15	Proteintech	Rabbit	Poly	55407-1-AP	1:500
Vash1	Abcam	Rabbit	Mono	ab199732	1:1000
Mapre1 (EB1)	Sigma-Aldrich	Rabbit	Poly	E3406	1:500
GAPDH	GenScript	Mouse	Mono	A01622-40	1:2000
H3	Abcam	Mouse	Mono	ab24834	1:3000
Anti-mouse	LI-COR	Donkey	Poly	925-32212	1:10000
Anti-rabbit	LI-COR	Donkey	Poly	925-68073	1:10000

### Mass Spectrometry (MS) Sample Preparation

To quantify the relative changes of multiple proteins of interest in the total protein lysate, aliquoted diluted total protein lysate samples were thawed at 22°C. One part 4x loading buffer freshly supplemented with 10% v/v ΒΜΕ was mixed with three parts total protein lysate. The final samples were heated to 100°C for 8 min. The heated samples were cooled to 22°C, centrifuged briefly, vortexed thoroughly, and loaded 50 μL/sample onto precast protein gels (Bio-Rad: 4561034). Protein gel electrophoresis was carried out under constant voltage of 110 V for the Mini gels for 1.5 h. The resolved protein gel was stained with Coomassie blue (Bio-Rad: 1610435) using the provided protocol. After the destaining of the gel, the 50 kDa bands were carefully excised and stored in deionized water at 4°C until further processing.

The gel bands were destained with 100 mM ammonium bicarbonate/acetonitrile (50:50). The bands were reduced in 10 mM dithiothreitol/100 mM Ammonium bicarbonate for over 60 min at 52°C; the bands were then alkylated with 50 mM iodoacetamide/100 mM ammonium bicarbonate at 22°C for 1 h in the dark. The proteins in the gel bands were digested with enzymes while incubating overnight at 37°C; different enzymes such as trypsin, Chymotrypsin, and Glu-C were used according to protein sequences. The supernatants were transferred and kept in fresh tubes. Additional peptides were extracted from the gel by adding 50% acetonitrile/1% TFA and incubated for 10 min on a shaker. The supernatants were combined and dried. The dried samples were reconstituted using 0.1% formic acid for MS analysis.

### MS Analysis Using Nano-LC-MS/MS

Peptides were analyzed on a Q-Exactive HF (Thermo Fisher Scientific) attached to an Ultimate 3000 rslcnano system (Thermo Fisher Scientific) at 400 nL/min. Peptides were eluted with a 55 min gradient from 5% to 32% ACN (25 min) and 90% ACN over 5 min in 0.1% formic acid. Data-dependent acquisition mode with a dynamic exclusion of 45 s was enabled. One full MS scan was collected with a scan range of 350–1,200 *m*/*z*, resolution of 70 K, maximum injection time of 50 milliseconds, and AGC of 1 × 10^6^. Then, a series of MS2 scans were acquired for the most abundant ions from the MS1 scan (top 12). Ions were filtered with charges 2–4. An isolation window of 2 m/*z* was used with quadruple isolation mode. Ions were fragmented using higher-energy collisional dissociation (HCD) with a collision energy of 27%. Orbitrap detection was used with a scan range of 140–2000 m/*z*, resolution of 30 K, maximum injection time of 54millisecondsmilliseconds, and AGC of 50,000.

### MS Data Analysis

Proteome Discoverer (Thermo Fisher Scientific, version 2.4) was used to process the raw spectra. Default search parameters were used, including precursor mass tolerance of 10 ppm, fragment mass tolerance of 0.02 Da, enzymes specific cleavage, and up to 2 mis-cleavage. Carbamidomethyl [C] was set as a fixed modification, while Oxidation [M] and Acetylation [N-terminal and K] were set as variable modifications. The target-decoy approach was used to filter the search results, in which the false discovery rate was less than 1% at the peptide and protein levels. For measuring the relative protein abundances, all the chromatographic data were aligned and normalized to peptide groups and protein abundances, missing values were imputed and scaled. The normalized protein abundance values from four Ctrls, 5 PE, and four Iso 4-days mice were used in the subsequent analysis. Since the different tubulin isoforms share multiple homologous regions, only unique peptides that are unambiguous to each isoform were used to calculate protein abundance. The unique peptides acquired for the analyzed isoforms ranged from 1–13 peptides and the full suite of peptide and protein groups used in the analysis can be found in the public proteomic repository as outlined in the data availability statement. Statistical analyses were performed on the calculated protein abundances as described below.

### Total RNA Extraction

Frozen aliquoted cardiac tissue obtained from similar locations of the heart was pulverized finely using a liquid nitrogen-cooled mortar and pestle. 500 μL of ice-cooled RNAzol (Molecular Research Center: RN 190) was added to the pulverized tissue and immediately homogenized using the handheld homogenizer until visible chunks of tissues were dissociated. 200 μL of molecular grade water was added to the sample; the sample was vortexed and incubated for 15 min at 22°C. After processing of all samples, the samples were then centrifuged at 12000 g for 15 min at 22°C. 550 μL of the clear supernatant was carefully removed and transferred into a fresh tube. 550 μL of isopropanol was then added to the supernatant, vortexed, and incubated for 10 min at 22°C. The samples were centrifuged at 16000 g for 10 min at 22°C, and the resulting supernatants were discarded. The visible RNA pellets were washed in 75% ethanol in molecular grade water three times. The undried RNA pellets were resuspended in 30 μL of RNase free water. The total RNA concentrations, and 260/230 and 260/280 ratios were determined using NanoDrop ND-1000 Spectrophotometer (NanoDrop Technologies). The RNA samples were stored at -80°C until further analysis.

### NanoString nCounter Analysis

Total RNAs from 37 samples were analyzed. The concentration of the total RNA was reassessed using NanoDrop spectrophotometer. The quality of the total RNA was assessed using the Agilent 4200 TapeStation (Agilent Technologies). Only samples that were pure as defined by OD 260/280 and 260/230 ratios >1.8, and integrity RIN value >8.0 were used in the study. 100 ng of total RNA per sample for tubulin and hypertrophy panels or 200 ng of total RNA per sample for tubulin autoregulation panel was used for the subsequent step. Hybridization between the target mRNA and reporter-capture probe pairs was performed for 18 h at 65°C using Mastercycler Pro S Thermal Cycler (Eppendorf) according to the manufacturer’s protocol. Post-hybridization processing was carried out on a fully automated nCounter Prep Station (NanoString Technologies) liquid-handling robotic device using the High Sensitivity setting. For image acquisition and data processing, the probe/target complexes were immobilized on the nCounter cartridge that was then placed in the nCounter Digital Analyzer (NanoString Technologies) as per the manufacturer’s protocol with FOV set to 555. The expression level of a gene was measured by counting the number of times the probe with a unique barcode, which was targeted against that gene, was detected. The barcode counts were then tabulated in a comma-separated value (.csv) format.

### NanoString nCounter Data and Statistical Analysis

The raw digital counts of expressions were exported into nSolver Analysis software (NanoString, version 4.0) for downstream analysis. The data was analyzed in nSolver using the Nanostring Analysis and Advanced Analysis software packages. The background of the data was thresholded using the geometric means after removing negative control values that are three-times higher than the rest. The data was then normalized using the geometric means of the positive controls, after removing “F” if the value is too close to background, and the three housekeeping genes (Gapdh, Rpl4, Tbp). Without removing low count values, the Bonferroni-corrected differentially expressed gene (DEG) analysis of the normalized data was computed using Treatment as covariates. For tubulin autoregulation panel, raw counts were exported, and statistical analyses were carried as outlined below.

### Statistical Analysis

Graphing and statistical analyses were performed using OrginPro 2019 software (OriginLabs). First, the normality of the data was determined using the Shapiro-Wilk test. For comparison of data distributions whose normality cannot be rejected at 0.05 level, the calculated probability of the means (p) between the control and the experimental group was calculated using the two-tailed two-sample Welch-corrected student’s t-test. For comparison of data distributions whose normality is rejected at 0.05 level, the p-value between the control and the experimental group was calculated using the two-tailed two-sample Kolmogorov-Smirnov test. For significance level, we used the Bonferroni-corrected significance cut-off of *p* < 0.025 denoted by *; ** represents *p* < 0.01 and *** represents *p* < 0.001. *p*-values to two significant figures were reported for 0.05 < *p* < 0.025. For all bar graphs, the bar represents mean and the whisker represents +1 SEM. For all box plots, the bolded line represents mean, and the whiskers represent ±1 standard error of mean (SEM).

## Results

### Tubulin Autoregulation is Operant in the Heart and Induced in Heart Failure

Despite the importance of microtubule proliferation in cardiac pathology, any role of tubulin autoregulation has not been examined. We utilized a previously established strategy to test for autoregulation by measuring the relative abundances of pre-spliced (i.e. intron-containing) and spliced (i.e. those without introns) tubulin mRNAs (Gasic et al., 2019). Using this approach, one can detect transcriptional regulation of a target through correlated changes in intronic and exonic mRNA levels (y = x in Figure 1E, for example), whereas post-transcriptional autoregulation would only affect exonic mRNA (shift along the y-axis of Figure 1E). To study autoregulation in an isoform-specific fashion, we designed NanoString nCounter probes for direct and unique detection of either intronic or exonic regions of individual tubulin isoforms. To determine if autoregulation is operant in heart muscle cells, we treated isolated mouse cardiomyocytes for 6 h with colchicine, a microtubule depolymerizing agent predicted to trigger autoinhibition (decrease in only exonic species) by increasing free tubulin, or taxol, a microtubule polymerizing agent predicted to trigger autoactivation (increase in only exonic species) by shifting free tubulin into the polymerized pool. Consistently, depolymerization significantly reduced the amount of exonic but not intronic mRNA across most tubulin isoforms, while polymerization increased the amount of exonic tubulin mRNA (Figures 1D,E). This data serves as the first demonstration that autoregulation is operant in the cardiomyocyte and that it regulates the majority of tubulin isoforms.

Next, we tested whether tubulin autoregulation can partially explain the discrepancy in mRNA and protein levels observed in HF. To this end, we designed a separate Nanostring probe set against introns and exons of human tubulin isoforms and probed RNA extracted from 35 cardiac samples from 12 non-failing donors and 23 patients with advanced heart failure. Figures 1F,G shows the relative intron and exon abundances for all tubulin isoforms that could be readily detected at the intron, exon and protein level (Chen et al., 2018) (Figures 1A,B). In failing hearts, the majority of tubulin isoforms showed reduced exonic relative to intronic levels, indicative of active autoinhibition across most isoforms. An exception is *TUBA1A*, the only isoform that demonstrated significant transcriptional induction; consistently *TUBA1A* is also being the only isoform to show both increased and correlated mRNA and protein levels in this HF population (Figures 1A,B). Of additional note, *TUBB4B* is by far the most abundant β-tubulin isoform expressed in the heart, and it exhibits robust autoinhibition in HF, yet maintains increased protein abundance. Taken together this data indicates that in HF, elevated tubulin protein triggers persistent autoinhibition of tubulin mRNA. The maintained elevation in tubulin protein may be explained by significantly increased tubulin stability/lifetime.

However, there remains no explanation as to how the heart achieved the increased tubulin protein in the first place or whether autoregulation plays any role in the establishment of the increased tubulin mass observed in pathological cardiac remodeling. To better understand this, we employed mice models of cardiac hypertrophy that allows us to explore the early roles of tubulin transcription, autoregulation, and stability.

### Acute Adrenergic Agonism Induces Anatomic and Transcriptional Cardiac Remodeling

**FIGURE 2:**
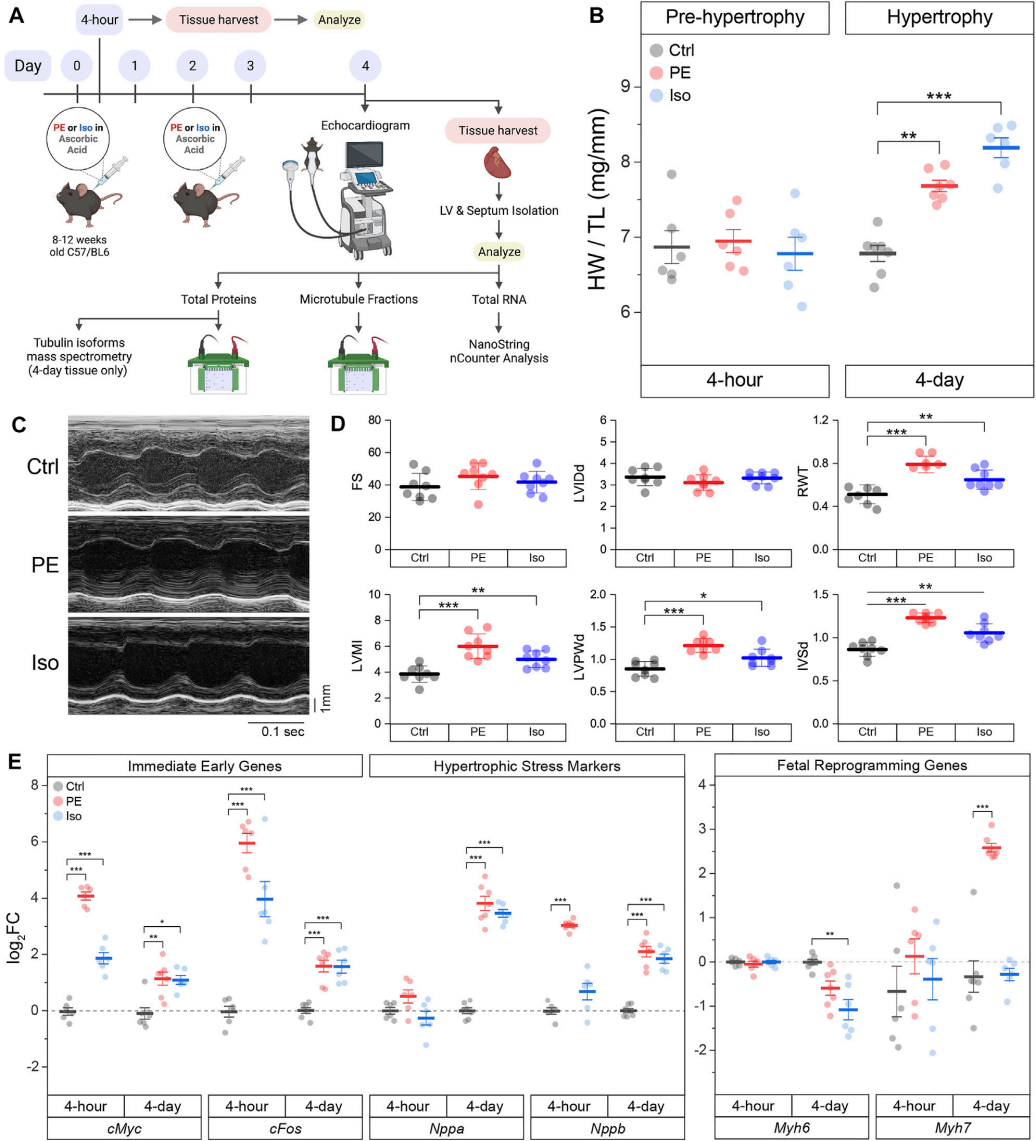
Acute α- or β- adrenergic stimulation induces cardiac hypertrophy. **(A)** Graphical scheme of the experimental plan. **(B)** Heart-weight/Tibia length (HW/TL) data of mice 4-h (*n* = 6) or 4-days (*n* = Ctrl:7, PE:7, Iso:6) following 10 mg/kg/injection of phenylephrine (PE) or 5 mg/kg/injection of isoproterenol (Iso). **(C)** Representative echocardiographic M-mode images of 4-days mice hearts. **(D)** Quantification of relevant echocardiographic parameters: FS = Fractional Shortening, LVIDd = Left-Ventricular Internal Diameter at end diastole, RWT = Relative Wall Thickness, LVMI = Left-Ventricular Mass Index, LVPWd = Left-Ventricular Posterior Wall thickness at end diastole, IVSd = InterVentricular Septal thickness at end diastole (*n* = 8). **(E)** Relative log_2_fold-change of nCounter mRNA counts of Immediate Early Genes (IEGs), hypertrophic stress markers, and genes of fetal reprogramming (*n* = 4 h: 6, 4 days: Ctrl:7, PE:7, Iso:6). For all box plots, whiskers represent ± 1SEM and bolded-lines represent mean. For **(B, D)**, * represents p-value from Welch-corrected two-tailed two-sample student’s t-test < 0.025, ** represents *p* < 0.01, and *** represents *p* < 0.001. For **(E)**, * represents Bonferroni adjusted (for 45 genes) p-value < 0.025 (Bonferroni-corrected for two comparisons), ** represents adj_*p* < 0.01, and *** represents adj_*p* < 0.001 (see Methods for more statistical details).

To determine how the microtubule network remodels during the development of cardiac hypertrophy, we characterized the myocardial cytoskeleton at two time points in two mouse models of adrenergic agonist-induced hypertrophy (Scarborough et al., 2021) (Figure 2A). A 4-h post-injection time point was chosen to capture a stage when hearts were exposed to hypertrophic stimuli but have not yet hypertrophied, and a 4-days post-injection time point was chosen to capture a stage when hearts had demonstrably hypertrophied. As expected, no change in heart-weight-to-tibia-length (HW/TL) was observed 4 h after injection of either phenylephrine (PE) or isoproterenol (Iso) compared to vehicle control (Ctrl) (Figure 2B, left). When mice were given a second injection on day 2 and hearts were collected on day 4, we observed a consistent cardiac hypertrophy with both PE and Iso (Figure 2B, right). To assess left-ventricular remodeling and function, we performed echocardiography on the 4-days hypertrophy animals. We observed consistent evidence of concentric hypertrophy upon both PE and Iso treatment, with elevated left-ventricular (LV) mass and increased wall and septal thickness (Figures 2C,D). Neither group exhibited evidence of decompensation toward HF, with no evidence of ventricular dilation or depressed contractility, indicating a compensated, concentric hypertrophy in response to acute adrenergic agonism.

We further validated our models using NanoString nCounter to assess transcriptional markers of cardiac remodeling in the hearts of PE and Iso treated mice. Using direct RNA counting of 42 transcripts sorted into immediate early genes (IEGs), hypertrophy-related genes, and fibrosis-related genes, we analyzed differentially expressed genes (DEGs) in the septa and LV of our time-matched control and experimental groups. We hypothesized that IEGs would be upregulated after 4-h of adrenergic stimulation, followed by upregulation of canonical markers of hypertrophic remodeling after 4 days. Consistent with this hypothesis, we observed robust upregulation of the canonical IEGs—*cMyc* and *cFos*—in both PE and Iso treated mice at 4-h (Figure 2E and Supplementary Figure S2), followed by induction of stress markers—*Nppa* and *Nppb*—at 4-days, along with markers of fetal reprogramming including myosin isoform switching (reduced *Myh6:Myh7* ratio).

The full complement of DEGs in each experimental group at the 4-h and 4-days time points are depicted in Supplementary Figure S2. At both 4-h and 4-days after adrenergic agonism, we observed upregulation of hypertrophy-related gene *Fhl1* (Friedrich et al., 2012), and fibrosis-related genes *Ctgf* (Hayata et al., 2008) & *Vcan* (Vistnes et al., 2014). Additional potentially relevant DEGs included the upregulation of *Col4a1* (Steffensen and Rasmussen 2018) and *Timp1* (Barton et al., 2003), and the downregulation of *Agrn*(Bassat et al., 2017; Baehr et al., 2020), among others.

### The Microtubule Network Is Rapidly Detyrosinated Upon Hypertrophic Stimulation

**FIGURE 3:**
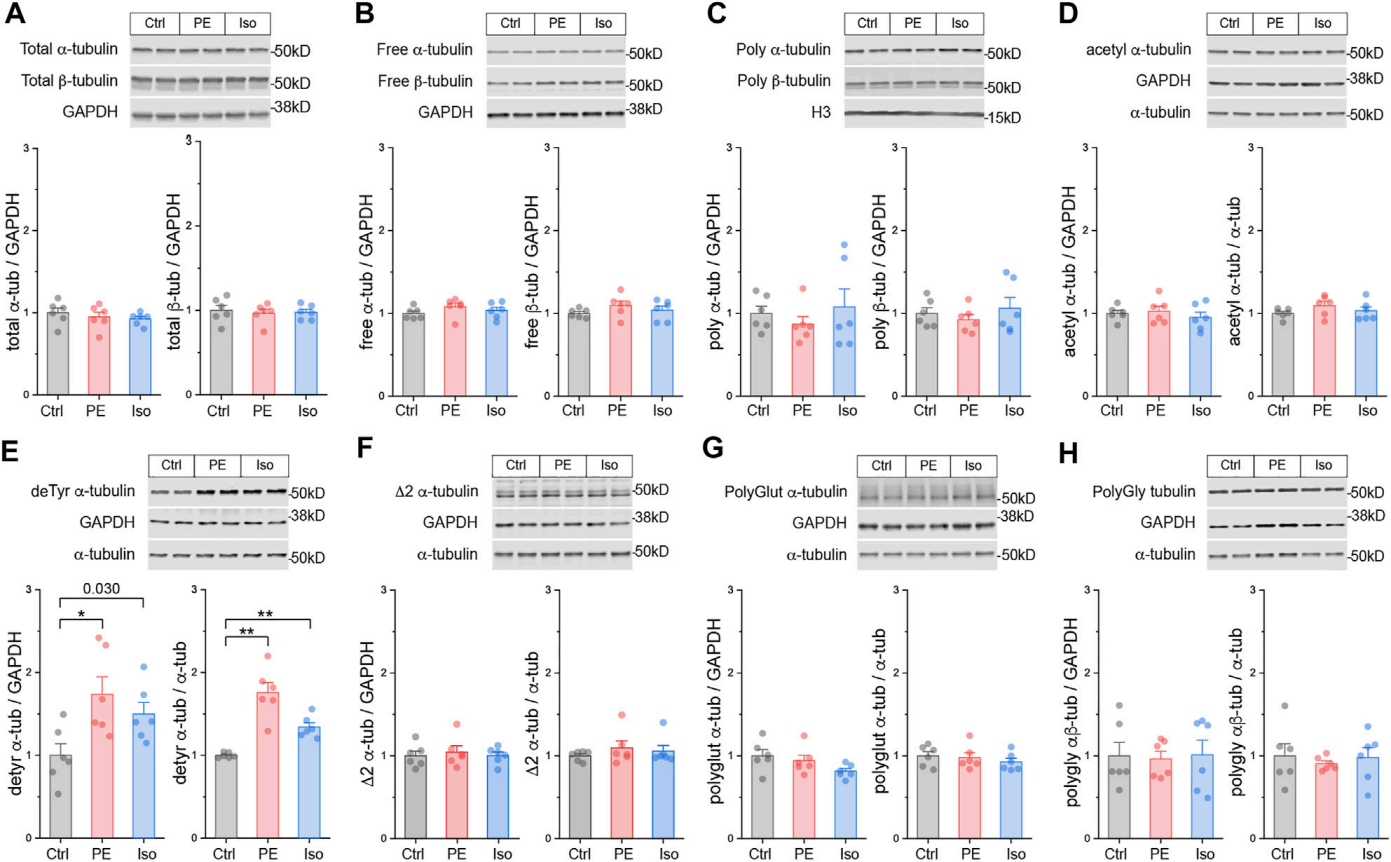
The microtubule network is rapidly detyrosinated upon hypertrophic stimulation. Representative immunblots and relative fold-change of α-tubulin (left) and β-tubulin (right) in **(A)** total proteins, **(B)** free or **(C)** polymerized -tubulin fractions (*n* = 6). Representative immunoblots with technical duplicate lanes and relative fold-change using GAPDH (left) or α-tubulin (right) as loading controls for **(D)** acetylated α-tubulin, **(E)** detyrosinated α-tubulin, **(F)** Δ2 α-tubulin, **(G)** polyglutamylated α-tubulin, & **(H)** polyglycylated pan-tubulin (*n* = 6). For all bar plots, whiskers represent + 1SEM and bar represents mean. For all graphs, * represents p-value from Welch-corrected two-tailed two-sample t-test < 0.025 (Bonferroni-corrected for two comparisons), ** represents *p* < 0.01, and *** represents *p* < 0.001.

Having validated the 4-h and 4-days models, we examined microtubule network remodeling in these contexts. We first determined whether the total αβ-tubulin content and free vs. polymerized tubulin pools are altered in the pre-hypertrophic (4-h) state. We observed no significant differences in these metrics of total tubulin content or fractionation at this early time point (Figures 3A–C).

We next determined whether tubulin is rapidly post-translationally modified upon hypertrophic stimulation. We immunoblotted for the five best-studied PTMs using validated antibodies: acetylation, detyrosination, polyglutamylation, polyglycylation, and Δ2 tubulin. Polyglutamylation, polyglycylation, and Δ2 are well characterized in cilia, flagella, and the brain (Paturle-Lafanechère et al., 1994; Aillaud et al., 2016), but they have not been studied in the heart. Detyrosination and acetylation, which occur predominantly on polymerized microtubules, are common markers of stable, long-lived microtubules, and of microtubule damage and repair-stabilization processes (Portran et al., 2017; Xu et al., 2017) respectively.

At the 4-h time point, we did not observe any significant differences in either the absolute (PTM/GAPDH) or the relative (PTM/α-tubulin) amounts of acetylation, polyglutamylation, polyglycylation, or Δ2 tubulin (Figures 3D,F–H). Surprisingly, we did observe robust induction of the absolute and relative amounts of detyrosination in both PE and Iso treated groups (Figure 3E). These data suggest that within 4 h of hypertrophic stimulation, prior to other overt changes in tubulin mass, microtubules are rapidly detyrosinated, which may serve as an early driver of microtubule stabilization.

### Post-Translationally Modified Microtubules Proliferate During the Establishment of Cardiac Hypertrophy

We next characterized microtubule network remodeling at day 4, concurrent with cardiac hypertrophy. We probed the three tubulin pools and immunoblotted for α-tubulin, β-tubulin, acetylation, detyrosination, polyglutamylation, polyglycylation, Δ2 as described above.

**FIGURE 4:**
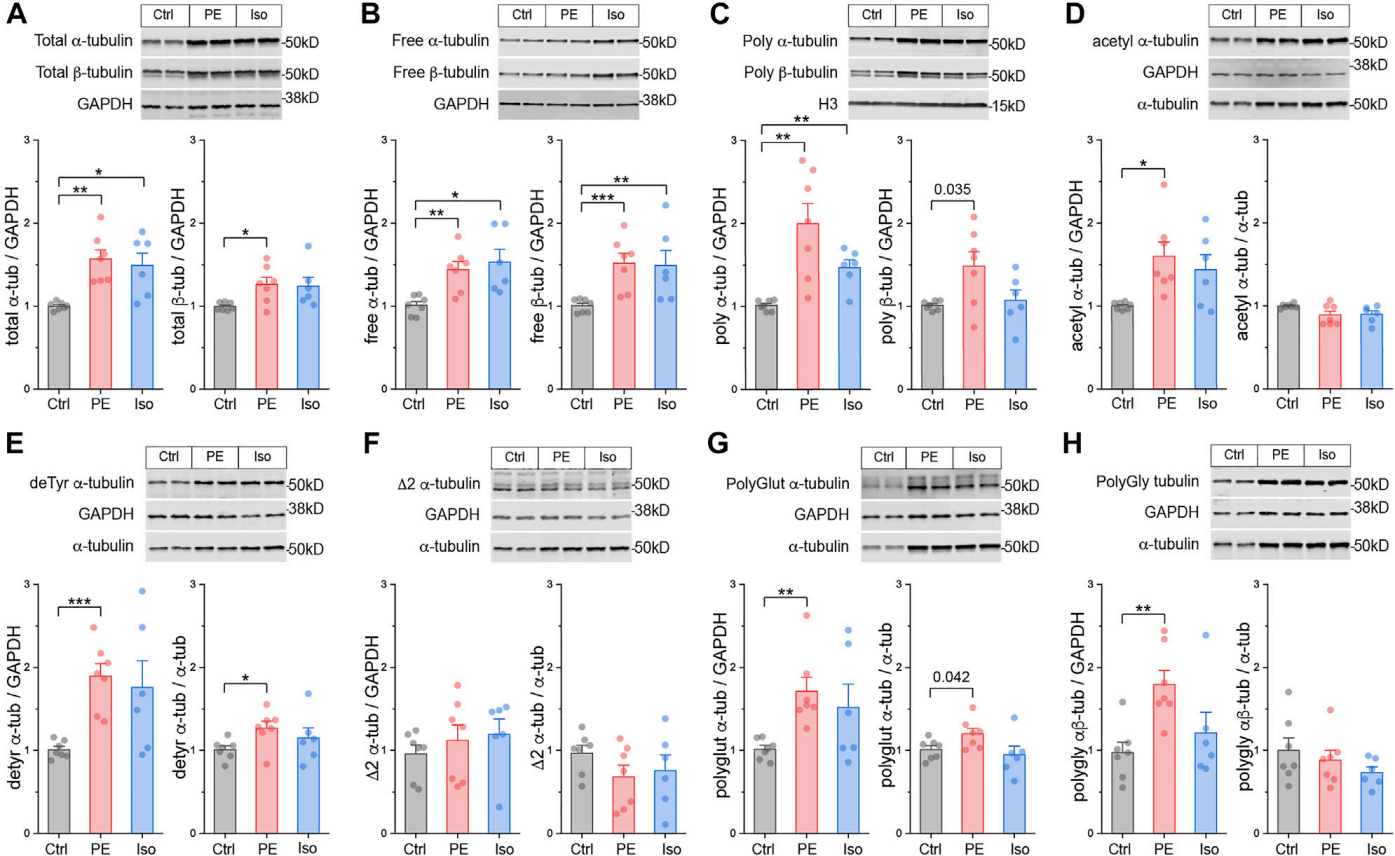
Total tubulin content increases and the microtubule network densifies during hypertrophy. Representative immunoblots and relative fold-change of α-tubulin **(left)** and β-tubulin (right) in the **(A)** total proteins, **(B)** free, or **(C)** polymerized -tubulin fractions (*n* = Ctrl:7, PE:7, Iso:6). Representative immuno- blots with technical duplicate lanes and relative fold-change using GAPDH (left) or α-tubulin (right) as loading controls for **(D)** acetylated α-tubulin, **(E)** detyrosinated α-tubulin, **(F)** Δ2 α-tubulin, **(G)** polyglutamylated α-tubulin, & **(H)** polyglycylated pan-tubulin (*n* = Ctrl:7, PE:7, Iso:6). For all bar plots, whiskers represent + 1SEM and bar represents mean; * represents p-value from Welch-corrected two-tailed two-sample t-test < 0.025 (Bonferroni-corrected for two comparisons), ** represents *p* < 0.01, and *** represents *p* < 0.001.

At this stage we observed increased free, polymerized, and total αβ-tubulin protein in the hearts of PE and Iso-treated mice (Figures 4A–C). In the PE group, the ratio of free:polymerized α-tubulin decreased (Supplementary Figure S3A), consistent with enhanced microtubule stability. In PE-treated mice, we observed increases in the absolute amounts of acetylation, polyglutamylation, and polyglycylation, and in the absolute and relative amounts of detyrosination. Iso treated mice showed a similar trend for each PTM, but of reduced magnitude and greater variability (Figures 4C–H). Taken together, these data indicate that during cardiac hypertrophy tubulin content increases, the polymerized network densifies, and there is a proportionally increased abundance of post-translationally modified microtubules with a modest enrichment of detyrosination.

**FIGURE 5:**
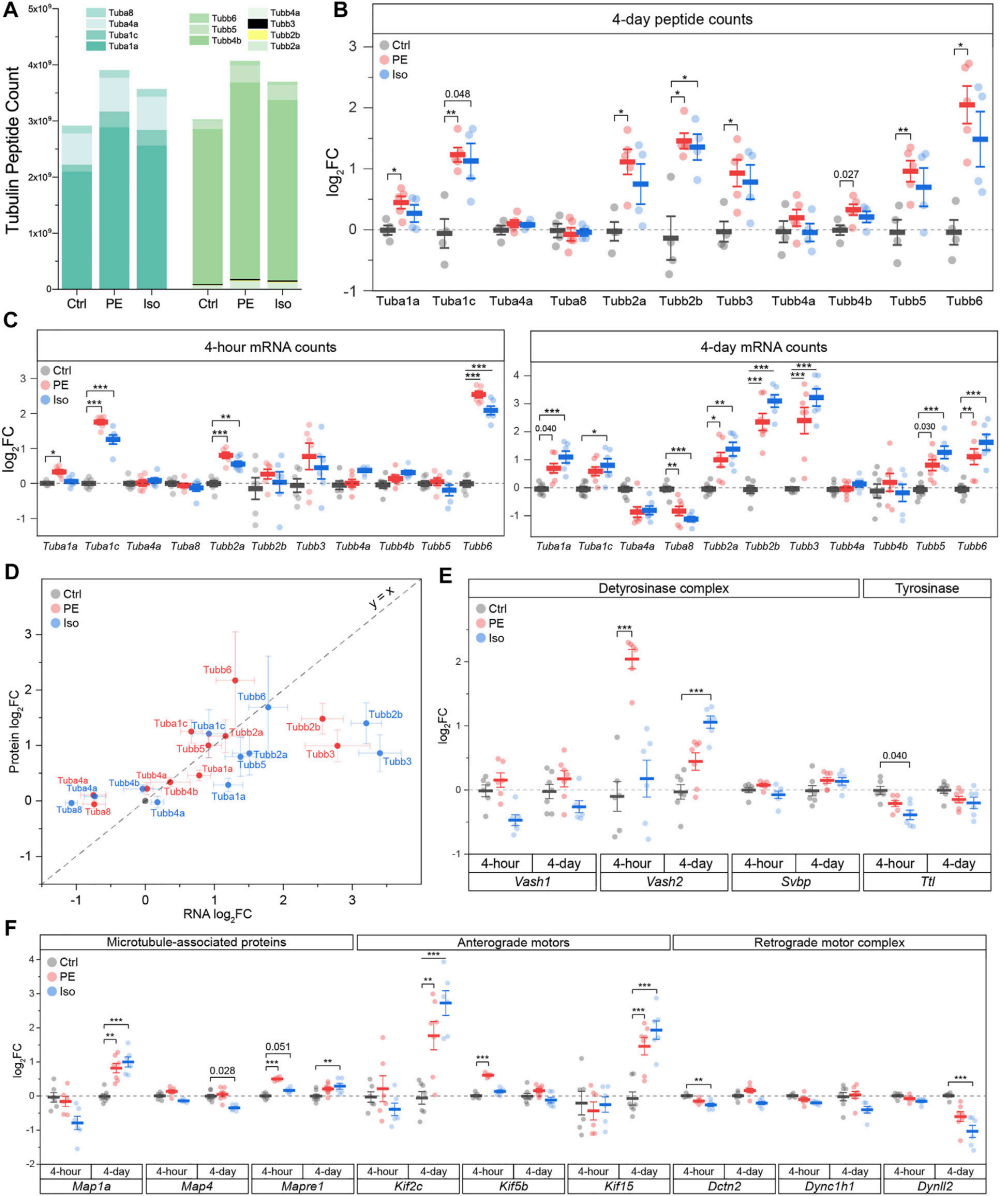
Differential expression of tubulin isoforms, modifying enzymes and MAPs during the onset and establishment of hypertrophy. **(A)** MS counts of unique peptides of detectable αβ-tubulin isoforms at 4-days timepoint. For all following box plots, whiskers represent ± 1SEM and bolded line represents mean. **(B)** Relative log_2_fold-change of αβ-tubulin isoforms peptide counts at 4-days (*n* = Ctrl: 4, PE: 5, Iso: 4); * represents p-value from Welch-corrected two-tailed two-sample t-test on non-log data <0.025 (Bonferroni-corrected for two comparisons), ** represents *p* < 0.01, and *** represents *p* < 0.001. **(C)** relative log_2_fold-change of nCounter mRNA counts of detectable tubulin isoforms at 4-h (left) (*n* = 6) and 4-days timepoints (right) (*n* = Ctrl:7, PE:7, Iso:6); * represents Bonferroni adjusted (for 50 genes) p-value < 0.025, ** represents adj_*p* < 0.01, and *** represents adj_*p* < 0.001 (see Methods and Materials for more statistical details). **(D)** Scatter-plot of log_2_fold-change of mRNA on x-axis and log_2_fold-change of protein on y-axis at 4-days timepoint; whiskers represent ± 1SEM and y = x represents a proportionate change between mRNA and peptide. Relative log_2_fold-change of nCounter mRNA counts of **(E)** detyrosinase complex and tyrosinase, & **(F)** MAPs, anterograde, & retrograde motors at 4-h (*n* = 6) and 4-days timepoints (*n* = Ctrl:7, PE:7, Iso:6); * represents Bonferroni adjusted (for 50 genes) p-value < 0.025, ** represents adj_*p* < 0.01, and *** represents adj_*p* < 0.001 (see Methods for more statistical details).

We next sought to determine how specific tubulin isoforms contribute to the increase in tubulin content observed at 4-days. To this end, we utilized mass spectrometric (MS) analysis of the total tubulin pool. We observed that the predominant α- and β-tubulin isoforms of murine LV were Tuba1a and Tubb4b, respectively (Figure 5A). Each of these predominant isoforms were modestly increased upon PE and Iso treatment. We also determined the relative changes of all detectable tubulin isoforms and observed significant increases in Tuba1a, Tuba1c, Tubb2a, Tubb2b, Tubb3, Tubb5, and Tubb6 (Figures 5A,B). Of note, Tuba4a—the only tubulin isoform that is synthesized in its detyrosinated form—was clearly not increased upon hypertrophic stimulation. This indicates that the early increases in detyrosination are not due to increased synthesis of Tuba4a, and instead likely due to altered activity of the enzymes of the tyrosination cycle. Tubb6 exhibited the highest degree of upregulation with a ∼4-fold increase upon PE treatment; this is notable as Tubb6 induction has been causally implicated in microtubule network reorganization in Duchenne Muscular Dystrophy (Randazzo et al., 2019). Despite significant upregulation of multiple low abundance isoforms, the overall composition of the total tubulin pool is largely conserved at this stage of hypertrophic remodeling (Figure 5A).

### Transcriptional Analysis of αβ-Tubulin Isoforms, Tubulin Modifying Enzymes, and MAPs During the Induction and Establishment of Hypertrophy

We next examined the contribution of transcriptional changes to the protein and network level microtubule remodeling at the 4-h and 4-days timepoints. To this end, we utilized NanoString analysis of total RNA using another set of 47 genes that includes tubulin isoforms, tubulin modifying enzymes, and MAPs.

While tubulin protein content was unchanged 4-h after adrenergic stimulation, we noted significant upregulation of several tubulin transcripts with both PE and Iso treatment at this stage, including *Tuba1c, Tubb2a and Tubb6*, with additional and more robust upregulation of *Tubb2b and Tubb3* by day 4 (Figure 5C). Consistent with proteomics assessments, *Tuba4a* and *Tuba8* were either unchanged or even downregulated upon PE and Iso treatment.

Regardless of the directionality of response, specific tubulin isoforms generally responded similarly to either adrenergic stimulus (Figure 5C). Further, in contrast to what was observed in advanced HF, transcript levels were also well-correlated with protein abundance across most isoforms at the 4-days time point (*R* ^2^ = 0.38, slope = 0.20, *p* = 1.4e-4) (Figure 5D). Consistent with protein expression lagging transcriptional regulation, the four isoforms (Tuba4a, Tuba8, Tubb2b, Tubb3) that displayed the greatest deviation in the change in the mRNA relative to the change in the protein levels (i.e., located furthest away from the y = x line when plotting log_2_FC of mRNA vs protein levels) were transcripts that showed delayed regulation; these isoforms were unchanged after 4-h but differentially expressed by 4-days. Consistent upregulation at the transcript and protein level was seen for Tuba1c, Tubb2a, Tubb2b, Tubb3, and Tubb6. Combined with the early upregulation of tubulin transcripts, this data indicates that increased tubulin mRNA at least partly underlies the isoform-specific increase in tubulin protein, and therefore tubulin mass, that is necessary for hypertrophic remodeling (Sato et al., 1997; Tsutsui et al., 1999; Scarborough et al., 2021).

We noted several additional transcriptional changes of tubulin modifying enzymes and MAPs that may bear relevance to cardiac remodeling and warrant further investigation (Figures 5E,F; Supplementary Figure S4). These include: (1) *Vash2,* which encodes a tubulin detyrosinase, exhibited the greatest differential expression among the 47 assessed transcripts at the 4-h PE time point; this may contribute to the robust early induction of detyrosination in this group (2) Early upregulation of *Kif5b* after 4-h in PE (Tigchelaar et al., 2016), which encodes the primary transport kinesin heavy chain 1 implicated in mRNA transport during myocyte growth (Scarborough et al., 2021); (3) upregulation of *Mapre1* in both PE and Iso at 4-h*,* which encodes a member of microtubule associated protein RP/EB family of +TIP tracking protein that guides microtubule growth; (4) Robust upregulation of *Kif15* in both PE and Iso by 4-days, which encodes a kinesin family member implicated in stabilizing parallel growing microtubules; (5) induction of *Map1a* in both PE and Iso at 4-days, which encodes a stabilizing structural MAP.

We next sought to determine whether these mRNA changes were reflected at the protein level for targets for which we could obtain robust signal via western blot from validated antibodies. Generally, transcripts that were upregulated early at the 4-h time point such as *Kif5b* and *Mapre1* also appeared to be upregulated at the protein level by day 4, whereas transcripts that were unchanged or only upregulated later at 4-days (such as *Vash1* and *Kif15*), did not show protein level changes at day 4 (Supplementary Figures S3B,C).

All tubulin-associated transcript volcano plots (Supplementary Figure S4) were asymmetric, tending to show a greater degree of upregulated than downregulated genes, implying a generalized induction of a tubulin-associated program at 4-days. This was particularly evident in the PE groups, and with progressive upregulation from the 4-h to 4-days time point. There were, however, notable down-regulated transcripts. While kinesin isoforms, which encode plus-end directed anterograde motors, were generally upregulated in treated groups, transcripts encoding subunits of the dynein/dynactin minus-end directed motor (*Dynll2, Dync1h1, Dctn2*) were either downregulated or unchanged (Figure 5F). This preferential induction of anterograde motors would bias trafficking toward the microtubule plus-end and away from the minus-end, which has implications for directed cardiac growth and for autophagic flux, which requires minus-end directed transport (McLendon et al., 2014). We also noted the early downregulation of enzymes involved in the polyglutamylation cycle, such as cytosolic carboxypeptidase 5 (*Ccp5*) and TTL-like family members 1 and 5 (*Ttll1/5*), which were all reduced in PE and Iso at the 4-h time point (Supplementary Figure S4).

To determine the conservation of these tubulin-associated transcriptional responses across varied hypertrophic stimuli, we compared our data with publicly available RNA sequencing datasets from two separate studies that examined early time-points following pressure-overload and angiotensin II induced hypertrophy. While data is not available for all transcripts, transcripts that were consistently reported across studies demonstrate well-conserved transcriptional signatures at both early (hours) and later (days) timepoints (Supplementary Figure S5), including the consistent upregulation of most αβ-tubulin isoforms but with the notable downregulation of *Tuba4a* and *Tuba8*.

### Transcriptional and Autoregulatory Mechanisms Underlie Isoform-specific Increases in αβ-Tubulin mRNA

**FIGURE 6:**
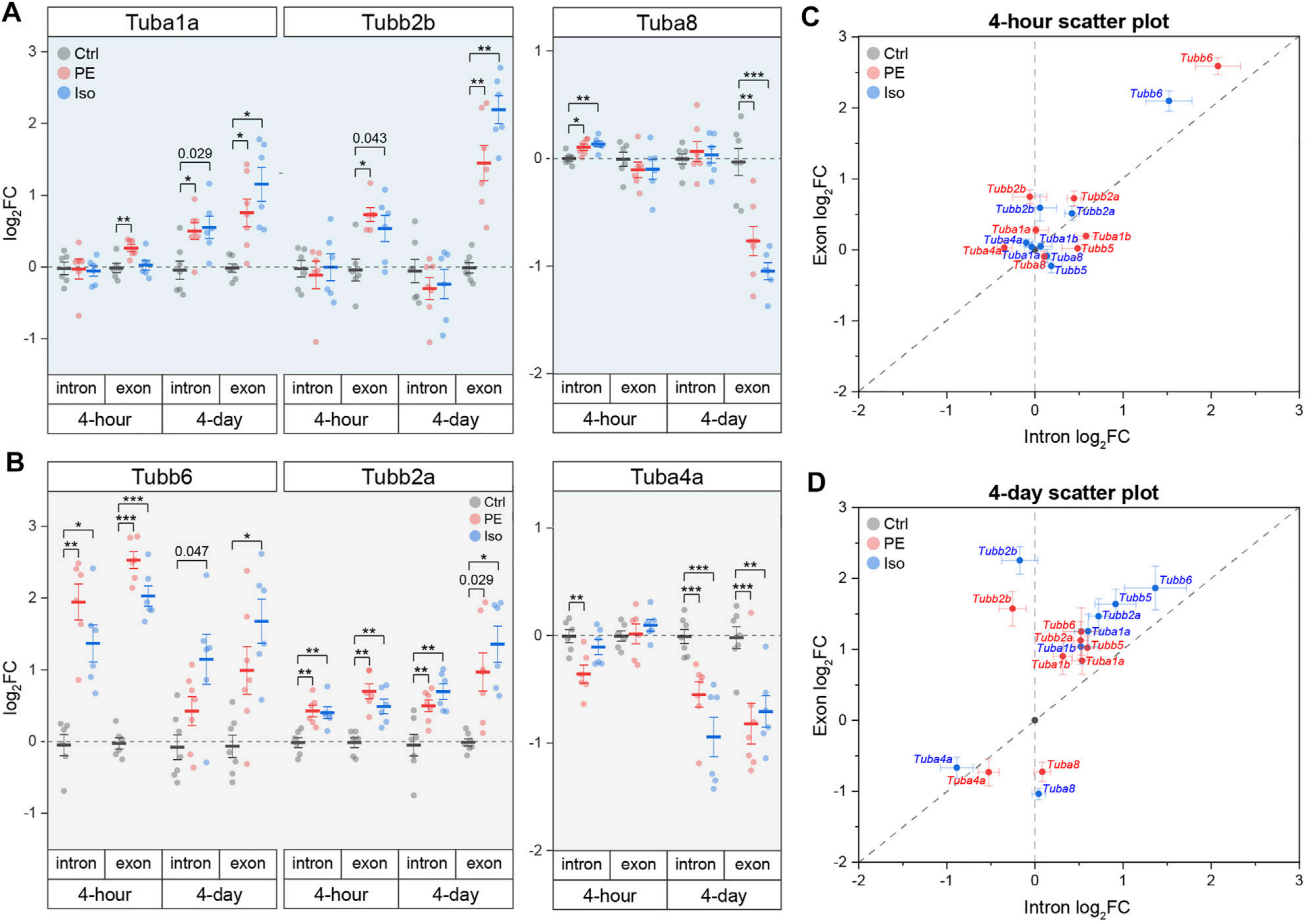
Tubulin isoforms are differentially regulated at the mRNA level through isoform-specific transcription and/or autoregulation during cardiac hypertrophy. Relative log_2_fold-change of mRNA counts of αβ-tubulin isoforms that are predominantly regulated through **(A)** autoregulation, or **(B)** transcription (n = 4 h: 6, 4 days: Ctrl:7, PE:7, Iso:6); * represents p-value from Welch-corrected two-tailed two-sample t-test on non-log data <0.025 (Bonferroni-corrected for two comparisons), ** represents *p* < 0.01, and *** represents *p* < 0.001. Scatter-plots of relative log_2_fold-change of intron on x-axis and exon on y-axis at **(C)** 4-h and **(D)** 4-days timepoints; whiskers represent ± 1SEM.

The above transcriptional and proteomic profiling indicates that the upregulation of tubulin mRNAs is an early driver of microtubule proliferation during the development of hypertrophy. This may arise from two non-exclusive mechanisms—(1) increased transcription or (2) decreased autoregulation (i.e., autoactivation). To differentiate between the two, we utilized the tubulin isoform and location -specific approach outlined above to interrogate the mechanism of tubulin upregulation during cardiac hypertrophy. Overall, we observed that by day 4 the exonic levels of almost all tubulin isoforms increased more than intronic levels, suggesting a generalized autoactivation of tubulin isoforms driven by microtubule stabilization (Figure 6). The most prominent cases of autoactivation are that of *Tubb2b*, whose increase in transcript level is solely through an increase in exonic species at both 4-h and 4-days, and *Tuba1a*, whose immediate response at 4-h was through an increase in exonic level with no change in intronic level (Figure 6A). Additionally, in a subset of the tubulin isoforms—*Tuba1b*, *Tubb2a*, *Tubb5*, and *Tubb6—*we observed robust increases in intronic levels that indicate direct transcriptional activation by the hypertrophic stimuli (Figures 6B and Supplementary Figure S6). Interestingly, despite a generalized upregulation and autoactivation of tubulin isoforms in the early stages of hypertrophy, *Tuba4a* and *Tuba8* are downregulated and autoinhibited, respectively. These data collectively show that αβ-tubulin mRNA is controlled in an isoform-specific and time-dependent fashion through both transcriptional and autoregulatory mechanisms to rewrite the tubulin code during cardiac remodeling.

## Discussion

In this work we combined transcriptomic and proteomic assessments of advanced heart failure samples and temporally well-defined murine models of cardiac remodeling to understand how a dense and modified microtubule network is achieved. Among other observations expanded upon below, we arrive at four primary conclusions: 1) tubulin autoregulation is operant in the heart and represses mRNA levels of tubulin isoforms in HF, contributing to the observed discrepancy between tubulin RNA and protein in HF; 2) the microtubule network is rapidly post-translationally detyrosinated within 4 h of a hypertrophic stimuli; 3) concomitantly, the abundance of tubulin mRNA is rapidly altered in an isoform-specific fashion through both transcriptional and autoregulatory mechanisms; 4) the time-dependent upregulation of discrete αβ-tubulin transcripts drives an increase in microtubule mass during cardiac hypertrophy.

Combining our work with past literature, we arrive at a sequential model for the formation of a proliferated and stabilized microtubule network in the remodeled heart (Supplementary Figure S7). Within hours of a hypertrophic stimuli and prior to detectable growth, the microtubule network is detyrosinated (Figure 3E). Our data indicate that this increase in detyrosination is likely due to transcriptional (Figure 5E) or post-translational (Yu et al., 2021) upregulation of the recently identified detyrosinating enzyme complex, as our data argues against alternative mechanisms such as increased Tuba4a expression (Figure 5C), increased polymerized or long-lasting microtubule substrate (Figures 3C,D), or decreased TTL expression (Supplementary Figures S3B,C). Detyrosination serves as a network stabilizer to protect microtubules from breaking down by regulating their interaction with effector proteins (Peris et al., 2009; Chen et al., 2021). Microtubule stabilization, in turn, shuttles free tubulin into the polymerized microtubule pool, triggering autoactivation that increases tubulin mRNA stability and translation. How autoregulation may achieve isoform-specificity is not understood, although indicated by our data (see *Tubb2b* vs. *Tuba8*, Figure 6). In concert with post-transcriptional upregulation of tubulin mRNA, increased transcription of several isoforms concomitantly increases tubulin mRNA. Independent of the mode of upregulation, tubulin mRNAs appear to be efficiently translated, as mRNA levels are well correlated with peptide abundance across tubulin isoforms (Figure 5D). As the stimuli persists and the heart enlarges, the newly translated tubulin is integrated into the microtubule network, resulting in increased microtubule mass and additional substrate for post-translational modifications (Figure 4).

Insights into tubulin isoforms in muscle biology have pointed towards the potential detrimental effects of specific isoforms in muscle pathologies; for example, TUBB6 is upregulated in dystrophic skeletal muscles, and it contributes to microtubule disorganization and altered muscle regeneration in muscular dystrophy (Randazzo et al., 2019). Elevated TUBA4A in human cardiomyopathy contributes to the increased detyrosination that impedes myocyte function (Chen et al., 2018; Schuldt et al., 2021). Strikingly, when we examine publicly available transcriptomic and proteomic data from chronically hypertrophied or failing human hearts (Figure 1A), we observe an inverse relationship between the transcript and protein levels of almost all αβ-tubulin isoforms. It is worth noting that TUBA8 is the lone tubulin transcript that is consistently *increased* in HF while the protein level is consistently *decreased*. Intriguingly, *Tuba8* was also the sole isoform to clearly escape autoactivation (and appear seemingly autoinhibited) during early hypertrophic remodeling (Figure 6A). We have no current explanation for how or why Tuba8 shows unique regulation in both settings. In contrast to this inverse relationship in HF, we observed that during the establishment of hypertrophy, transcript and protein levels are highly correlated, suggesting an uncoupling of transcript and protein levels that occurs later in the course of cardiac remodeling. Chronic, robust microtubule stabilization and increased tubulin lifetime could account for the stably elevated tubulin protein content despite persistent autoinhibition that we observe in HF.

Our analysis permits the temporal evaluation of several cytoskeletal- or hypertrophy- associated factors at distinct stages representing the onset and establishment of cardiac hypertrophy. Beyond the key conclusions listed above, several additional observations on cytoskeletal remodeling are of note. The association of the microtubule network with motor proteins such as kinesins alters its mechano-biochemical properties as well as its density. As an example, Kif15 (kinesin-12) has been shown to cross-link nearby parallel microtubules, causing them to bundle, and subsequently decreases the catastrophic events of dynamic microtubules (Drechsler and McAinsh 2016). Interestingly, during both PE and Iso -induced hypertrophy, *Kif15* is upregulated, suggesting that *Kif15* could contribute to microtubule network densification. Kif5b (Kinesin-1), the predominant anterograde motor in the heart, was previously reported to be increased in PE induced-hypertrophy of neonatal rat ventricular cardiomyocytes (Tigchelaar et al., 2016). We observed similar and rapid increase in Kif5b transcript and protein levels in our hypertrophy models (Figure 5F, Supplementary Figure S3C). Kinesin-1 was recently identified to be required for the distribution of mRNA and ribosomes that enables cardiomyocyte hypertrophy (Scarborough et al., 2021), and past work indicates that kinesin-1 prefers to transport cargo along detyrosinated microtubule tracks (Kaul et al., 2014). Meanwhile, the dynein/dynactin retrograde motor protein complex (transcriptionally downregulated, Figure 5F), prefers tyrosinated microtubule tracks (Nirschl et al., 2016). Taking together, these observations suggest that the heart both rapidly induces its primary anterograde transport motor and remodels its preferred tracks in response to a hypertrophic stimulus.

Our findings indicate that rapid transcriptional, autoregulatory, and post-translational mechanisms remodel the microtubule network following a hypertrophic stimulus. Contextualized with past literature, these changes will support the ability of the microtubule network to bear increased mechanical load, facilitate mechanotransduction, and enhance transport of the translational machinery that is required for growth. In summary, the data points towards a concerted and adaptive response to establish hypertrophy, and we provide a resource for further investigation into the diverse roles of microtubules in cardiac remodeling.

## Data Availability

The Nanostring data presented in the study have been deposited to the GEO Omnibus repository with the accession number GSE194397. The mass spectrometry proteomics data have been deposited to the ProteomeXchange Consortium via the PRIDE partner repository with the dataset identifier PXD031797.

## Abbreviations

DEG, differentially expressed gene; HF, heart failure; HW, heart weight; Iso, isoproterenol; IEG, immediate early gene; PTM, post-translational modification; MAP, microtubule-associated proteins; αTAT1, α-tubulin acetyltransferase 1; VASH1/2, vasohibins 1 and 2; TL, tibia length; LV, left-ventricle; Ctrl, vehicle control; PE, phenylephrine.
