# Supplementary material for: Transcriptional, Post-Transcriptional, and Post-Translational Mechanisms Rewrite the Tubulin Code During Cardiac Hypertrophy and Failure
Source: Front Cell Dev Biol. 2022 Apr 1;10:837486. doi: 10.3389/fcell.2022.837486 (PMC9010559; doi:10.3389/fcell.2022.837486)
Supplement: Supplementary file 1 [file DataSheet1.pdf]

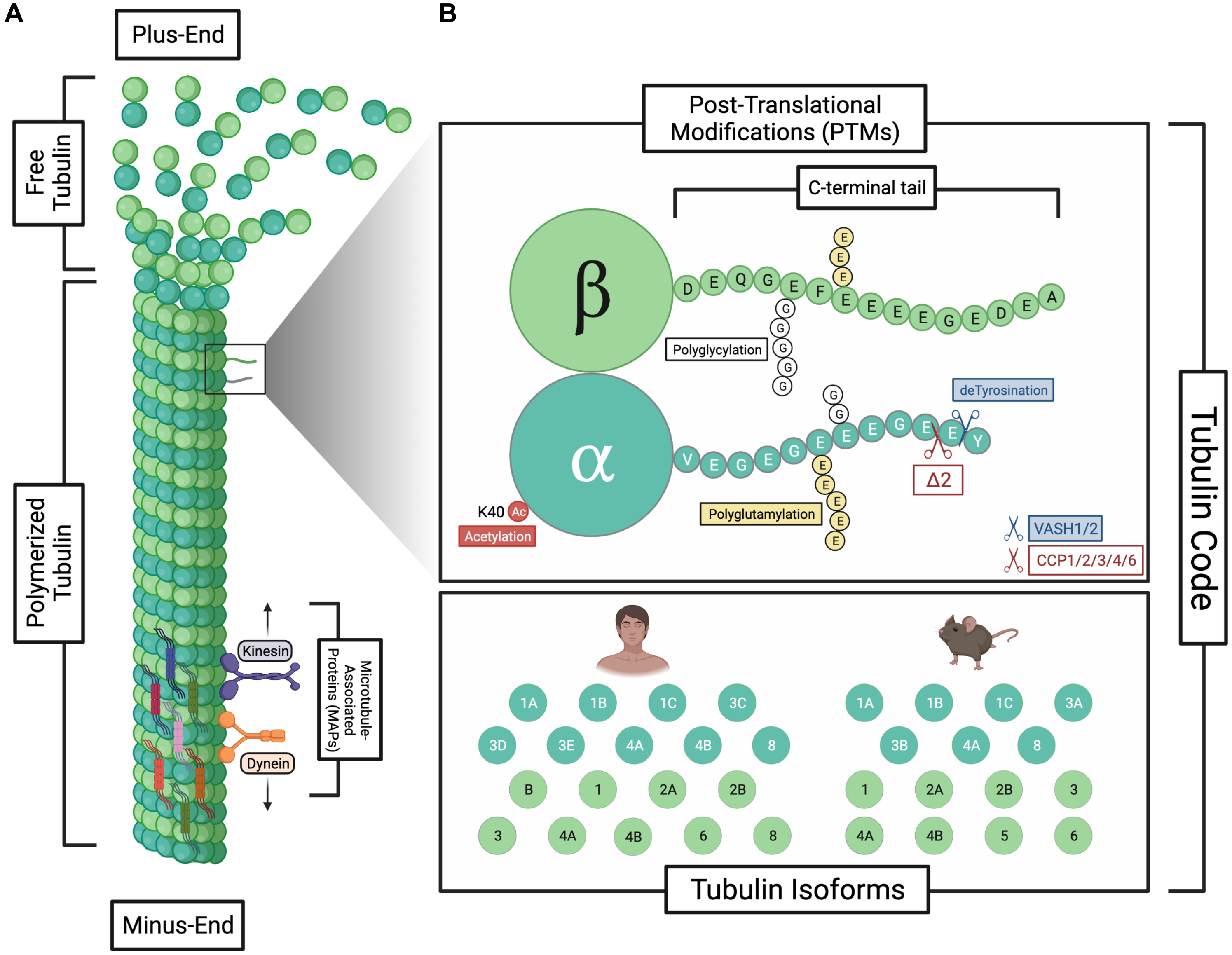

Supplemental Figure 1. Summary of the tubulin code. **(A)** Free & polymerized tubulins are in dynamic equilibrium. **(B)** The tubulin code is the permutation of the tubulin post-translational modifications & isoforms.

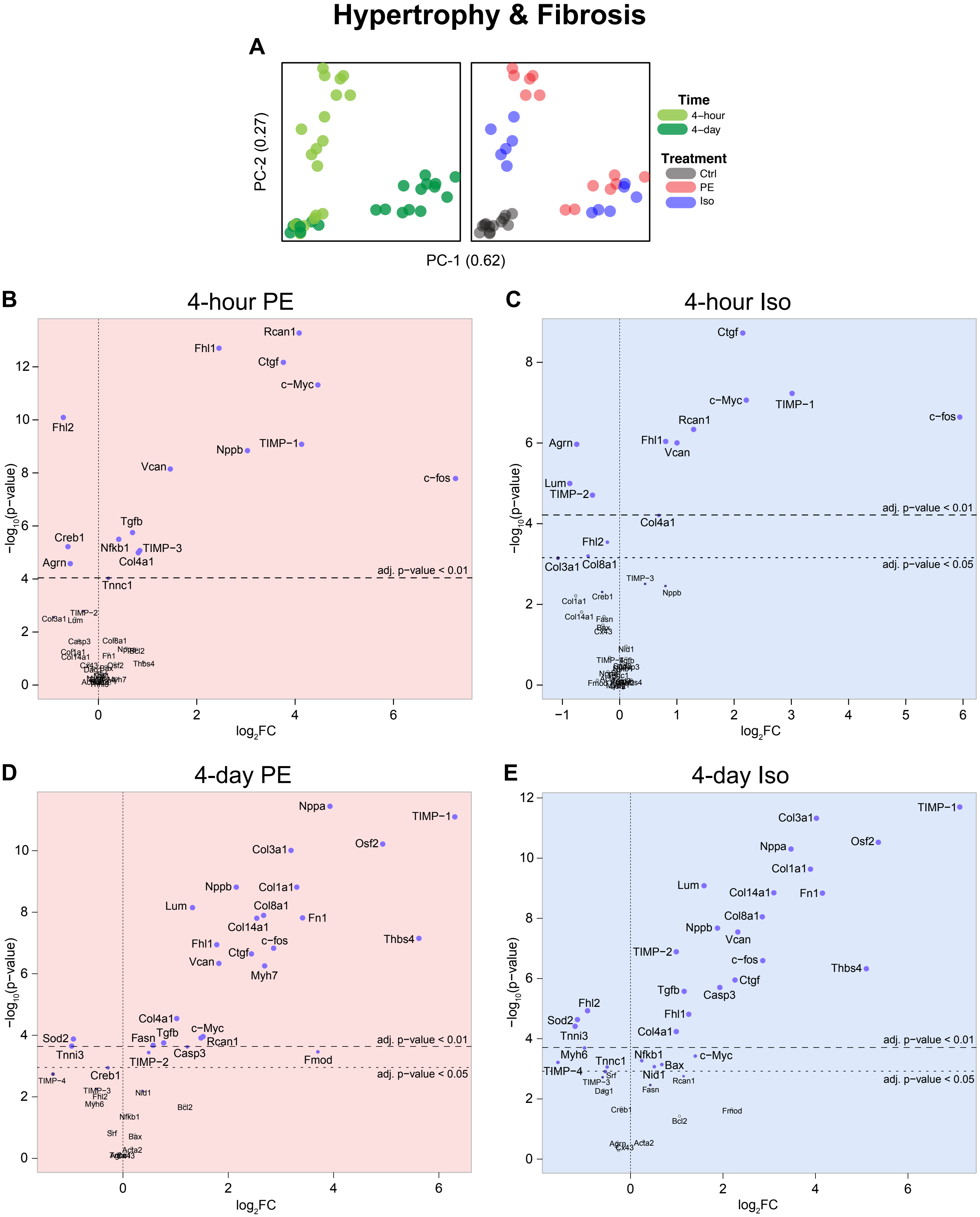

Supplemental Figure 2. (A) Principal component analysis (PCA) of nCounter mRNA counts from 4-hour and 4-day mice heart tissues using the hypertrophy & fibrosis panel. Volcano plots of all mRNA counts from the hypertrophy & fibrosis panel for **(B)** 4-hour PE, **(C)** 4-hour Iso, **(D)** 4-day PE, & **(E)** 4-day Iso. (n = 4h:6, 4d: Ctrl:7, PE:7, Iso:6)

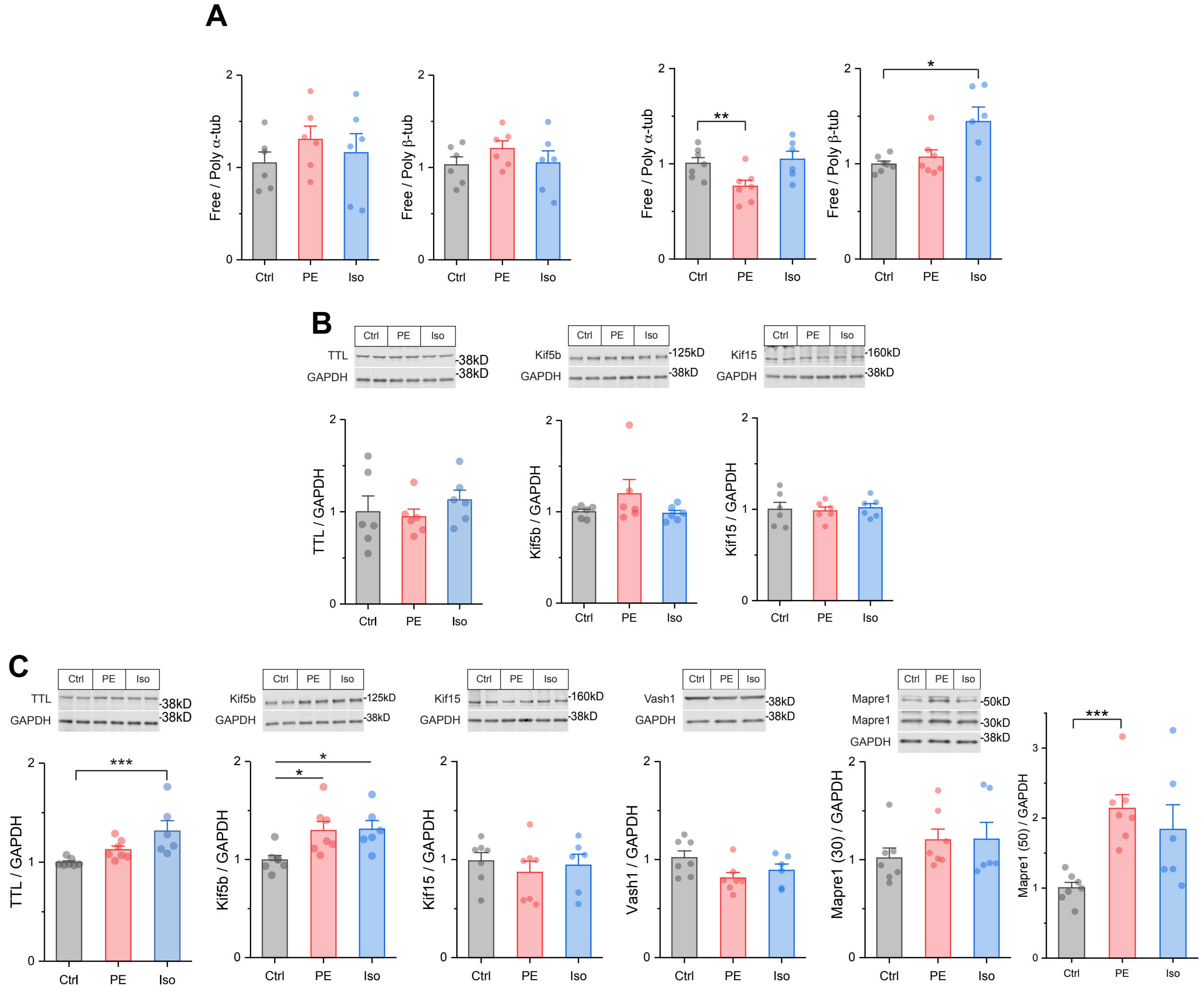

Supplemental Figure 3. (A) Free/Poly quantifications for α and β -tubulins at 4-hour **(left)** and 4-day **(right)**. **(B)** Western blot analyses of TTL, Kif5b, and Kif15 at 4-hour. (n: Ctrl, PE, Iso = 6) **(C)** Western blot analyses of TTL, Kif5b, Kif15, Vash1, and Mapre1 at 4-day. (n: Ctrl=7, PE=7, Iso=6). For Mapre1, we provided quantifications of two prominent bands at different molecular weights that were changed in the experimental groups: 30kDa band, which is the predicted molecular weight according to the amino acid sequence, and 50kDa band, which is also observed using the Anti-EB1 antibody produced in rabbit (Sigma: E3406) in certain cell types. For all graphs, * represents p-value from Welch-corrected two-tailed two-sample t-test < 0.025, ** represents p < 0.01, and *** represents p < 0.001.

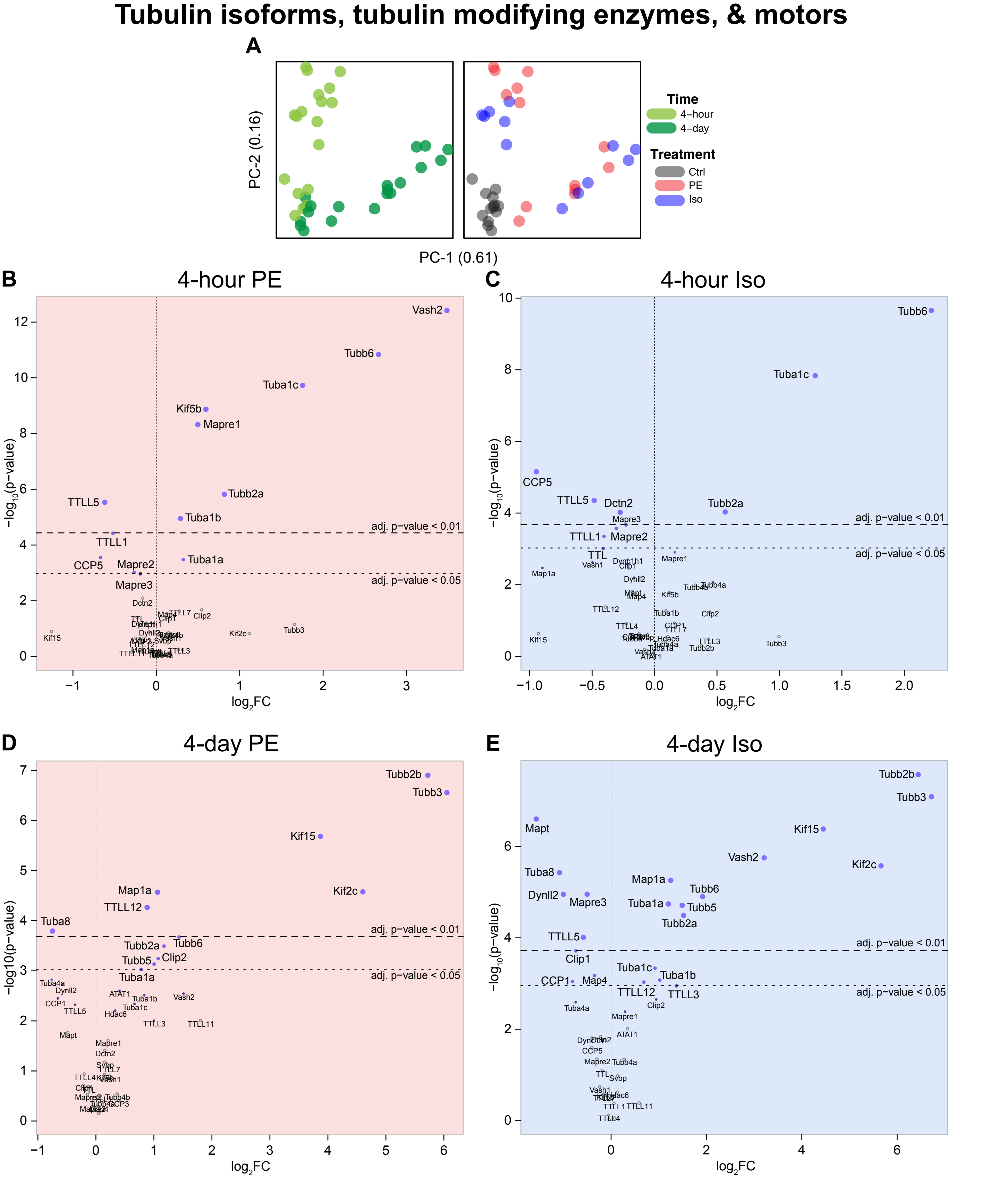

Supplemental Figure 4. (A) Principal component analysis (PCA) of nCounter mRNA counts from 4-hour and 4-day mice heart tissues using the tubulin panel. Volcano plots of all mRNA counts from the tubulin panel for **(B)** 4-hour PE, **(C)** 4-hour Iso, **(D)** 4-day PE, & **(E)** 4-day Iso. (n = 4h:6, 4d: Ctrl:7, PE:7, Iso:6)

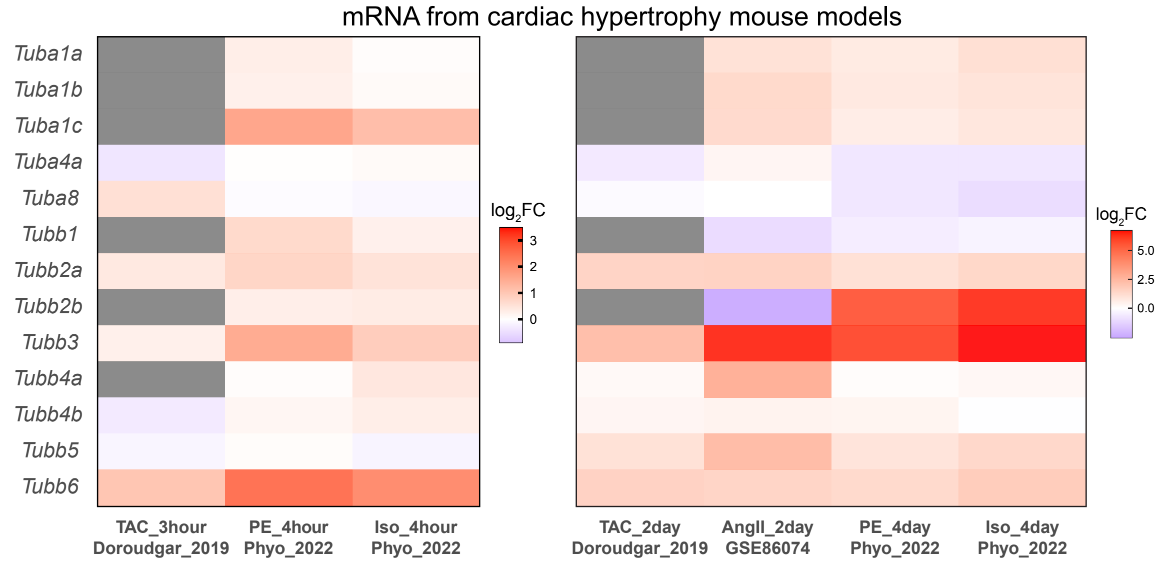

Supplemental Figure 5. Previously published & current studies' mRNA data of mouse αβ-tubulin isoforms following different pathological stimulations (Bottermann et al., 2018, Doroudgar et al., 2019).

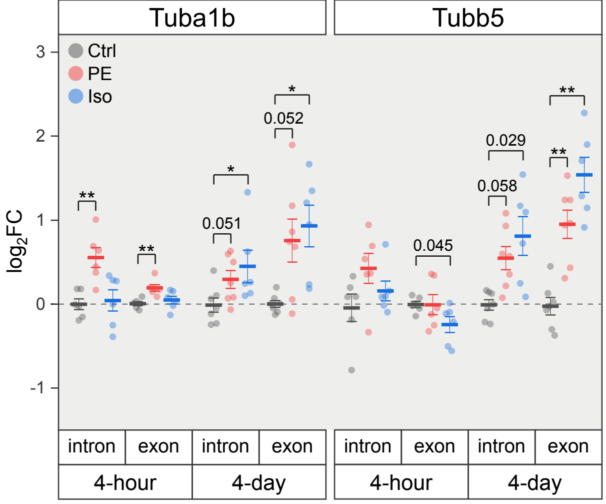

Supplemental Figure 6. Unspliced and spliced mRNA counts of Tuba1b and Tubb5 (n = 4h:6, 4d: Ctrl:7, PE:7, Iso:6); * represents p-value from Welch-corrected two-tailed two-sample t-test on non-log data < 0.025 (Bonferroni-corrected for two comparisons), ** represents p < 0.01, and *** represents p < 0.001.

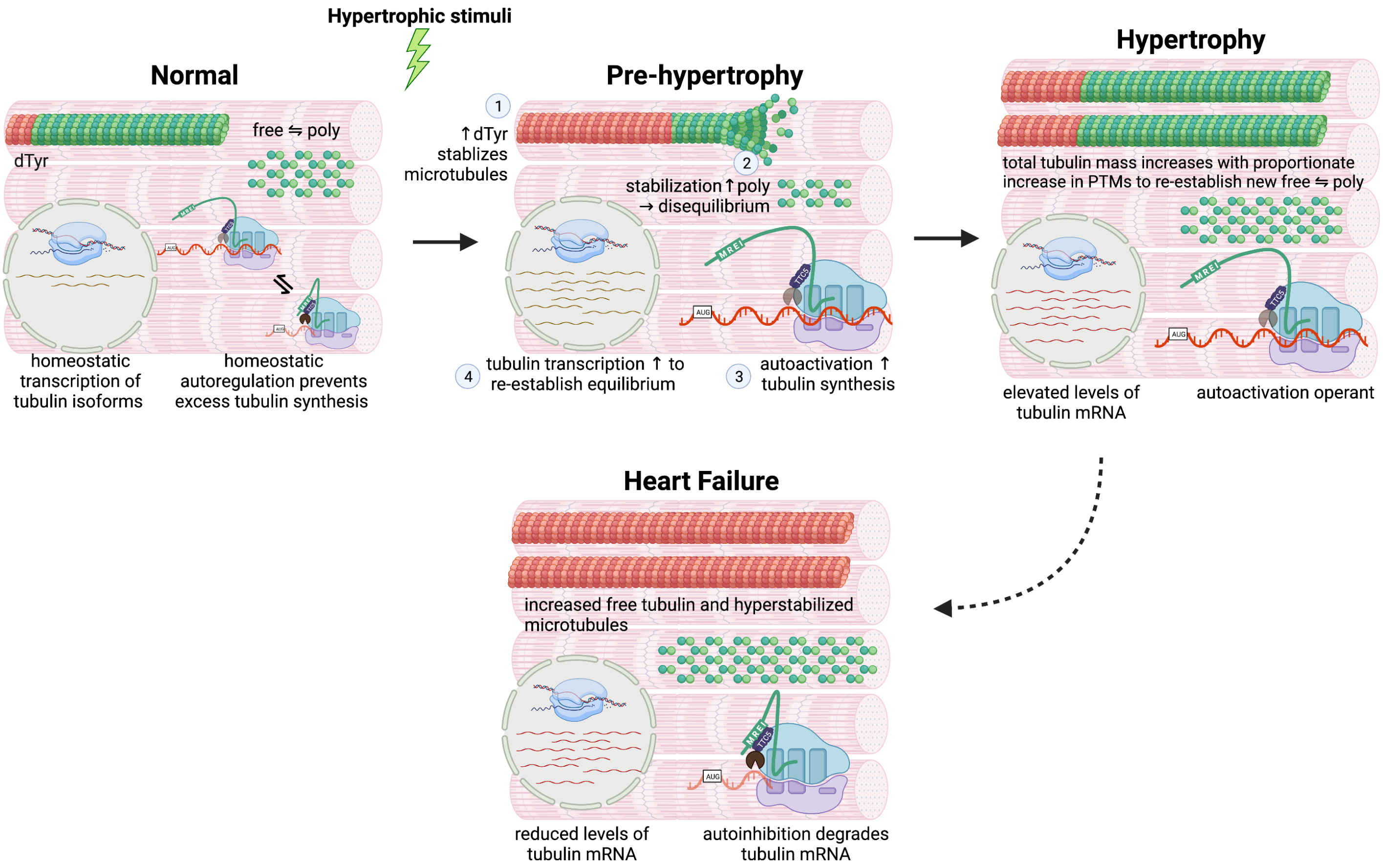

Supplemental Figure 7. Schematic model of the summary
